# Supplementary material for: The Influence of Depression on Biased Diagnosis of Premenstrual Syndrome and Premenstrual Dysphoric Disorder by the PSST Inventory
Source: Life (Basel). 2021 Nov 22;11(11):1278. doi: 10.3390/life11111278 (PMC8624424; doi:10.3390/life11111278)
Supplement: Supplementary file 1 [file life-11-01278-s001.zip › life-1399693-SI.pdf]

Article

The Influence of Depression on Biased Diagnosis of Premenstrual Syndrome and Premenstrual Dysphoric Disorder by the PSST Inventory

Andrzej Śliwerski * and Karolina Koszałkowska

Institute of Psychology, University of Łódź, 90-136 Łódź, Poland; karolina.koszalkowska@uni.lodz.pl

* Correspondence: andrzej.sliwerski@uni.lodz.pl

Table S1. Descriptive statistics for study sample.

	**Without depression**				With depression			
	**Without PMS/PMDD (*N* = 58)**	**PMS (*N* = 52)**	**PMDD (*N* = 14)**	Statistical test (df)	Without PMS/PMDD (*N* = 34)	PMS (*N* = 48)	PMDD (*N* = 9)	Statistical test
Mean age	24.79 (3.04)	24.77 (3.44)	23.21 (3.09)	F = 1.472(123) $p > 0.05$	23.82 (3.25)	24.29 (3.53)	22.33 (2.70)	F = 1.315(90) $P > 0.05$
Mean cycle length	28.60 (2.26)	28.73 (2.61)	28.64 (2.70)	F = 0.037(123) $p > 0.05$	28.50 (2.94)	28.83 (2.28)	28.00 (3.00)	F = 0.447(90) $P > 0.05$
CES-D	14.45 (9.88)	16.52 (8.84)	17.29 (9.29)	F = 0.910(123) $p > 0.05$	23.62 (12.24)	26.13 (11.61)	28.67 (14.41)	F = 0.779(90) $P > 0.05$
OC (n)<br>No OC (n)	13<br>45	11<br>41	1<br>13	χ^2 = 1.473(1) $p > 0.05$	7<br>27	16<br>32	1<br>8	χ^2 = 2.523(1) $p > 0.05$
Psychiatric treatment (n)	2	1	2	χ^2 = 3.717(1)	7	11	4	χ^2 = 2.286(1)

				$p > 0.05$				$p > 0.05$
Psychotherapy (n)	4	1	1	χ^2 = 1.992(1) $p > 0.05$	8	13	0	χ^2 = 2.986(1) $p > 0.05$

PMS/PMDD was diagnosed using prospective methods; CES-D - The Center for Epidemiologic Studies Depression Scale; OC - oral contraceptive; *SD*s are given in square brackets; χ^2 - Fisher's exact test;
